# Changes in vitelline and utero-placental hemodynamics: implications for cardiovascular development

**DOI:** 10.3389/fphys.2014.00390

**Published:** 2014-11-11

**Authors:** Kersti K. Linask, Mingda Han, Nathalie J. M. Bravo-Valenzuela

**Affiliations:** ^1^Department of Pediatrics, Morsani College of Medicine, Children's Research Institute, University of South Florida HealthSt. Petersburg, FL, USA; ^2^Perinatal Cardiology, Johns Hopkins All Children's HospitalSt. Petersburg, FL, USA

**Keywords:** embryonic heart, placenta, echocardiography, hemodynamics, Doppler ultrasound, blood flow, mouse, human pregnancy

## Abstract

Analyses of cardiovascular development have shown an important interplay between heart function, blood flow, and morphogenesis of heart structure during the formation of a four-chambered heart. It is known that changes in vitelline and placental blood flow seemingly contribute substantially to early cardiac hemodynamics. This suggests that in order to understand mammalian cardiac structure-hemodynamic functional relationships, blood flow from the extra-embryonic circulation needs to be taken into account and its possible impact on cardiogenesis defined. Previously published Doppler ultrasound analyses and data of utero-placental blood flow from human studies and those using the mouse model are compared to changes observed with environmental exposures that lead to cardiovascular anomalies. Use of current concepts and models related to mechanotransduction of blood flow and fluid forces may help in the future to better define the characteristics of normal and abnormal utero-placental blood flow and the changes in the biophysical parameters that may contribute to congenital heart defects. Evidence from multiple studies is discussed to provide a framework for future modeling of the impact of experimental changes in blood flow on the mouse heart during normal and abnormal cardiogenesis.

## Introduction

The human embryonic heart is a cylindrical beating tube already at 3 weeks of gestation. The earliest time at which human cardiac function can be visualized using ultrasound is ~6–7 weeks of gestation. Despite technological advances, echocardiographic patterns are still not sufficient to adequately visualize the human embryonic heart circulation at the early stages of cardiac development. The Doppler ultrasound patterns obtained during the earliest stages that have been analyzed during human gestation are similar to those observed for embryonic mouse heart function (Gui et al., 1996) and thus animal models remain useful to analyze *in vivo* embryonic heart function in normally developing embryos and in those displaying abnormal cardiac function. There has been an awareness from the early part of the last century (Thompson, 1917; Le Gros Clark and Medawar, 1947) that to interpret the generation of form and pattern of living organisms, one needs to define not only the genetic factors that determine the form of an organ, but also the influence of the physical forces to which the system is exposed in its normal developmental environment. So it is with organogenesis of the heart. In the last 10 years, a great deal has been learned about the role of hemodynamic force on cardiovascular development. Much of this knowledge has come from animal models and technological advances that have enabled researchers to analyze blood flow at earlier and earlier stages of cardiovascular development. The emphasis of many of these studies has been on modeling effects of intracardiac flow and how these forces are mechanotransduced. This review is to address how hemodynamics related to extra-embryonic circulations is associated with heart and vascular development.

Blood flow early in development is dependent on the yolk sac and development of the vitelline circulation. Later, as the placental circulation becomes functional, both extraembryonic circulations send blood to the developing heart. From studies from a number of groups, the results demonstrate that hemodynamic changes in the extraembryonic circulations, vitelline or placental, can alter normal heart development to induce cardiac anomalies. The objectives of this review are to provide (i) evidence from animal-based hemodynamic studies carried out on vitelline and placental circulations that have demonstrated a relationship with changes in normal heart and vascular development; (ii) evidence from human gestation using Doppler ultrasound parameters that demonstrate changes in placental hemodynamics are associated with altered human heart and vascular development; and lastly (iii) evidence from environmental exposure studies using animal models that demonstrate that fetuses displaying cardiac anomalies also show placental abnormalities.

The concept of an importance of the heart-placenta axis has been published earlier (Huhta and Linask, 2013; Linask, 2013). In this review emphasis is placed on studies that have dealt with the contribution of vitelline and placental circulations in relation to heart development, both normal and abnormal. In a recent editorial (Sliwa and Mebazaa, 2014) citing the work of Llurba et al. (2014) that is discussed below, the authors concluded that an “evaluation of the relationship between congenital heart defects (CHDs) and placenta-related complications should be explored in further research.” The intent for this review is to provide a brief overview of relationships that exist between CHDs and vitelline and placental blood flow for investigators involved in mathematical modeling of cardiac hemodynamic effects and mechanotransduction. It appears of benefit to this field to consider the role of placental blood flow, or even earlier, vitelline blood flow, and changes therein to understand the role of extraembryonic blood flow forces, often accompanied by hypoxia, in the formation of heart anomalies.

## Doppler ultrasound parameters of hemodynamics

### Introduction

During the early 1990's for analysis of abnormal mouse heart development we used a breeding scheme that generates the trisomic 16 mouse model (Miyabara et al., 1982; Epstein et al., 1985). In order to detect the one embryo in the litter that was developing a heart defect related to the trisomy condition and to carry out further analyses, we wanted viable embryos and also the ability to do longitudinal analyses on heart function of the same abnormally developing embryo within a litter. A more efficient, high-throughput manner was needed. For this we adapted for the first time for analysis of the embryonic mouse model the use of non-invasive Doppler echocardiography (echo) to evaluate each mouse embryo's heart function and demonstrated that echo can be used longitudinally to characterize normal cardiac functional changes during mouse gestation (Linask et al., 1993; Gui et al., 1996). The methodology worked surprisingly well with even using clinical ultrasound instrumentation with a 7.5 mHz transducer that was available to us for these early analyses. A detailed description of the methodology of doing the mouse exams was reported separately (Linask and Huhta, 2000). Shortly after our early studies, advances in non-invasive functional analyses using Doppler echocardiography were made possible with new ultrasound instrumentation for small animal imaging that was equipped with 40 mHz transducers (Srinivasan et al., 1998; Phoon and Turnbull, 2003; Phoon, 2006). A recent echocardiographic longitudinal assessment of embryonic and fetal mouse heart development using the high-frequency ultrasound system has been published that correlates the hemodynamic ultrasound analysis with the changes in mouse cardiac morphology during development (Hahurij et al., 2014). These ultrasound studies have allowed for a more sensitive measurement of physiological parameters of embryonic hemodynamics in the mouse model and at earlier time-points than previously was possible. It became apparent that the ability to do the analyses non-invasively allowed for more accurate quantitation of the physiological parameters than when carried out invasively (Gui et al., 1996; Keller et al., 1996; Phoon et al., 2000). We had also noticed in our early non-invasive analyses, that when we opened the maternal abdomen to carry out another echo exam on the same embryo, but this time directly, the embryonic heart rates had dropped and therefore quantitative reproducibility of the non-invasive data was not possible (Gui et al., 1996).

### Cardiac and placental ultrasound parameters summarized

Embryonic mouse heart rate increases as heart formation progresses during gestation. Accurate measurement of heart rate under normal physiological conditions is important because the other cardiac parameters of cardiac ejection, filling, and cardiac output are affected by the heart rate (Phoon et al., 2000). The normal Doppler characteristics of umbilical blood flow during mouse gestation indicated that the heart rate, peak blood flow velocities and velocity time integrals increased from E9.5 to 14.5, indicating increasing stroke volume and cardiac output (Gui et al., 1996; Phoon, 2001). Placental impedance was shown to decrease with gestation (Phoon et al., 2000). Similar results were reported in a subsequent study analyzing the developmental changes in hemodynamics of uterine artery, umbilical, and vitelline yolk sac circulation during mouse gestation (Mu and Adamson, 2006). It was also observed the initial heartbeats begin around 5 somite-stage and blood flow at the 8- to 10-somite stage (Ji et al., 2003). This would correspond to ~E8.5 in the mouse or ~33 h in the chick embryo. The first heartbeats are driven by the Na-Ca Exchanger (Koushik et al., 2001; Linask et al., 2001).

To obtain more information on placental function and development, non-invasive Doppler ultrasound of the umbilical artery can be used to provide an indirect analysis of umbilical blood flow and placental resistance (Maclennan and Keller, 1999; Phoon et al., 2000; Mu and Adamson, 2006). Using the C57Bl6 mouse model, we assessed different parameters of the fetal cardiac cycle and umbilical blood flow on E15.5 in response to acute exposure of embryos on E6.75 to different environmental factors (Han et al., 2009; Serrano et al., 2010). We routinely monitor embryos on E 15.5, because a four-chambered heart is now present with functional valves. During the echo exam, the pregnant mouse is anesthetized and core temperature monitoring is performed throughout the procedure to maintain body temperature. E15.5 corresponds to approximately week 10 of human gestation. The abnormal echocardiographic patterns relating to myocardial and valve defects upon environmental exposures have been published earlier (Han et al., 2009; Serrano et al., 2010). For venous hemodynamics, the presence of flow reversal during atrial contraction (A wave) in the ductus venosus and the presence of umbilical venous pulsations was used as the sign of abnormal flow/congestive heart failure (Huhta, 2005). The abnormal echo patterns relating to the myocardial and valve (atrioventricular and semilunar) defects after environmental exposures display variability, because not all embryos within a litter are at the same developmental stage. In our experimental exposure studies peak systolic velocity (PSV) and the end-diastolic velocity (EDV) were measured for embryos displaying cardiac defects within each experimental group and the results were averaged and compared to the same parameters in control embryos. The pulsatility (PI = (PSV − EDV)/time-averaged velocity, Gosling et al., 1991) and resistance (RI = PSV − EDV)/PSV, Giovagrandi et al., 1987) indices are discussed and reviewed below.

The elements of resistance to flow are those that are present in any other vascular bed, namely the number, length and diameter of the arteries, and the viscosity of the blood. The relationship is derived from Poiseuille's Law: Q = π r^4^/8n l, where Q is flow, r is radius, n is blood viscosity and l is vessel length. Although, a small change in vessel radius may be sufficient to increase the flow (4th power), vessel length and blood viscosity are factors that can influence the flow on a more minor scale. Therefore, when the umbilical arteries are narrow, they offer high resistance to flow as in first-trimester human pregnancy or in pathological conditions, such as when the umbilical vessels may be affected by an enviromental event or mother's vascular disease (pre-eclampsia and IUGR). Normally the pulsatility of arterial velocity waveforms increases when downstream resistance increases (Mu and Adamson, 2006).

## Animal-based hemodynamic studies carried out on vitelline and placental circulations demonstrating a relationship with changes in normal heart and vascular development

Development of functional circulatory systems for nutrient and oxygen exchange is imperative for the growth and survival of the embryo. Initial growth of the embryo is dependent on the vitelline circulation to the yolk sac. Abnormalities in yolk sac hemodynamics can result in embryonic malformations or lethality (Hogers et al., 1995, 1999). Later from embryonic day 10.5 (E10.5) during mouse gestation embryonic and fetal growth is dependent on umbilical blood flow via the chorioallantoic placenta. The hemodynamic relationships of these circulatory systems are discussed below.

### Yolk sac vitelline circulation

The period of trophoblast invasion that begins on embryonic day (E) 8.5 in the mouse coincides approximately with when the tubular heart first begins to beat in the mouse embryo (Phoon, 2001). Using Swiss–Webster mice, blood velocity was detected at E 8.5 in the vitelline artery supplying the yolk sac placenta in all embryos that were analyzed. It is to be noted that to image at these early developmental stages, a “semi-invasive” imaging technique was used by making a lower abdominal incision to access the embryo. This was done to optimize imaging of these small-sized embryos. Flow parameters under these semi-invasive conditions may not be completely physiological, but it is the best characterization that we currently can attain at this early developmental stage. Peak velocity increased almost sevenfold between E8.5 and E13.5 at which time it reaches a plateau and remains stable until term (Mu and Adamson, 2006). Positive EDV was detected in the vitelline artery only at E18.5. Despite higher peak velocities reached in the umbilical arteries, the yolk sac circulation is thought to be the most important source of nutrition for the embryo until E13.5, at which stage the umbilicoplacental circulation becomes paramount and the PSV has reached its plateau.

In human pregnancy, the vitelline artery and umbilicoplacental circulations are functional from the onset of the heart beat until the end of the organogenetic period (~9 weeks). The yolk sac in late mouse gestation appears to play a role in calcium transfer, immunoglobulin G, and amino acid transport. In the mouse both the placenta and yolk sac show hematopoiesis until E17 (Alvarez-Silva et al., 2003). In the human after organogenesis is completed during the first trimester, the yolk sac regresses and vitelline blood velocities are no longer detected.

**Figure 1:**
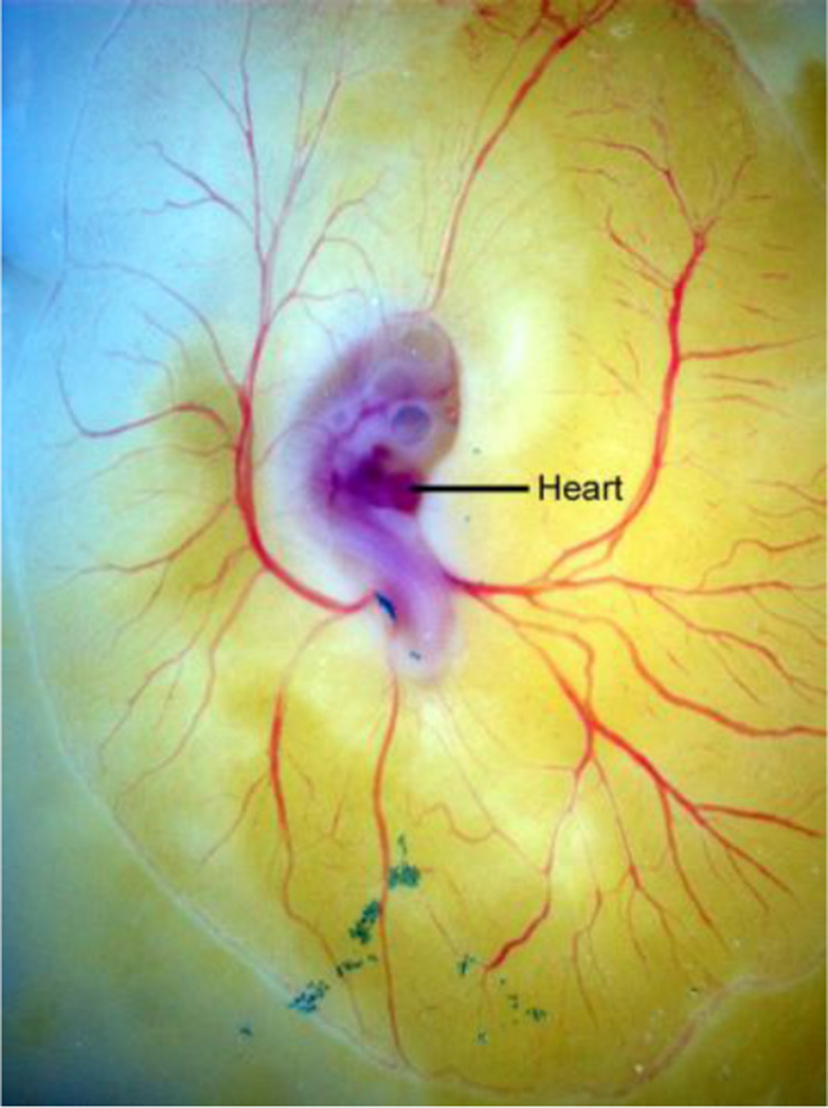
**Development of the avian extra-embryonic vasculature is shown displaying the normal modeling of blood vessels in the yolk sac (vitelline circulation) in an embryonic stage 14 quail embryo grown on yolk in shell-less culture**.

Concomitant with heart formation, blood and vascular vessels first form in the extraembryonic yolk sac (Figure 1 as observed in the avian model). Similarly by E8.5 a capillary network of endothelial-lined channels is evident in the mouse yolk sac. The vessels ultimately connect to the embryo, completing a circulatory loop between embryonic and extra-embryonic tissues. The first blood cells and endothelial cells are forming in yolk sac blood islands beginning at E7.0 in the mouse (Mcgrath et al., 2003). By E8.0 thousands of nucleated primitive red blood cells have formed within a vascular plexus in the yolk sac. Once the heart begins to beat, primitive erythroblasts originating in the yolk sac can be found in the embryo. The data suggest that a fully functional circulation is established after E10.0. The yolk sac vascular network remains a site of progenitor production even as the fetal liver later becomes a hematopoietic organ (Mcgrath et al., 2003).

#### Venous clip avian model and hemodynamics

Early studies analyzing the impact of vitelline hemodynamics on heart development were carried out using the chick model because of ease of experimental manipulation and visualization of results. Using a venous clip model whereby the right lateral vitelline vein was obstructed, the venous return and intracardiac laminar blood flow patterns were altered, with secondary effects observed on the mechanical load of the embryonic myocardium (Stekelenburg-de Vos et al., 2003). All hemodynamic parameters measured were seen to decrease acutely after the clipping. The effects were thought to be correlated with the observed cardiac malformations that formed in these embryos. Subsequently, in a pressure-volume loop analysis of the venous clipped model, it was demonstrated that clipping resulted in systolic and diastolic ventricular dysfunction. The ventricular functional changes preceded morphological abnormalities that became apparent in later developmental stages (Stekelenburg-de Vos et al., 2007). Shear stress is positively correlated to blood flow. Blood flow-related shear stress and circumferential strains have been associated with embryonic heart remodeling. These blood flow forces are transduced intracellularly via mechanotransducing molecular complexes, as for example, integrin-mediated complexes or tenascin C-associated (Linask and Vanauker, 2007; Garita et al., 2011).

In addition to the molecular sensors, an ultrastructural cellular adaptation is present on endothelial cells for mechanosensing called the primary cilium (Van der Heiden et al., 2006; Clement et al., 2009). The primary cilia are connected to the cytoskeletal microtubules and transmit information about the amount and direction of blood flow into the endothelial cells. During looping and heart remodeling from a C-shaped tube, the highest shear stress is experienced by the inner heart curvature. When shear stress is experimentally altered, this inner curvature region is highly affected and is associated with the development of CHDs (Hierck et al., 2008). The shear stress changes were shown to affect gene expression with KLF-2 being expressed in regions of highest shear stress and ET-1 and NOS-3 expressed in later stages in regions of shear stress (Groenendijk et al., 2004, 2005). We also observed primary cilia associated with embryonic cardiomyocytes within the embryonic myocardium during looping (Garita et al., 2011). In this optical coherence tomography (OCT) analysis of the looping chick heart, during each heartbeat the myocardial wall during filling pushes against the splanchnopleure and rolls for a short duration along the extraembryonic membrane, and then quickly moves away during systole. It was suggested the cardiomyocyte cilia have a role in sensing outside pressures of the splanchnopleural membrane to define spatially the embryonic heart looping process, as the loop tightens and changes with each heartbeat to eventually form the four-chambered heart.

#### Vascular remodeling and blood vessel structure formation

It appears that vascular remodeling and blood vessel structure formation in the mouse yolk sac are influenced also by hemodynamic forces (Lucitti et al., 2005, 2007). Using confocal microscopy and fluorescent dextran labeling, the studies demonstrated that the heart is a functional pump as soon as the myocardium begins to contract and that there is a significant period of plasma flow preceding the entry of erythroblasts into the circulation. Embryos with impaired cardiac contractility (homozygous *Myosin light chain2a* mice, *Mlc2a*^−/−^) displayed slow oscillatory flows instead of the normal pulsatile laminar-flow of normal embryos. The homozygote embryos displayed abnormal reduced plasma flow and delayed erythroblast circulation and defective vascular remodeling. When plasma flow rates were normal, but the erythroblasts were sequestered in the blood islands, lowering the hematocrit and decreasing shear stress, vessel remodeling was impaired indicating this process depends on erythroblast flow. The remodeling defects in the low-hematocrit embryos could be rescued by restoring the viscosity of the blood by injection of a high-molecular weight synthetic sugar (Lucitti et al., 2007). Expression of molecules involved in force transduction as eNOS was also restored to the endothelial cell membranes in response to the changes in mechanical force.

#### Flow forces and assembly of endothelial adherens junctions

It has been demonstrated in *in vitro* studies that the size of adherens junctions and tight junctions between endothelial cells closely match with the intercellular forces for the specific flow conditions (Ting et al., 2012). Laminar flow can increase cytoskeletal tension and disturbed flow decreases cytoskeletal tension in response to the shear flow conditions affecting in turn the assembly of cell-cell adherens junctions. Thus, the tractions forces have the ability to also regulate the barrier function of endothelial cells and tissue integrity.

### Umbilico-placental circulation

#### Organization of the placenta

**Figure 2:**
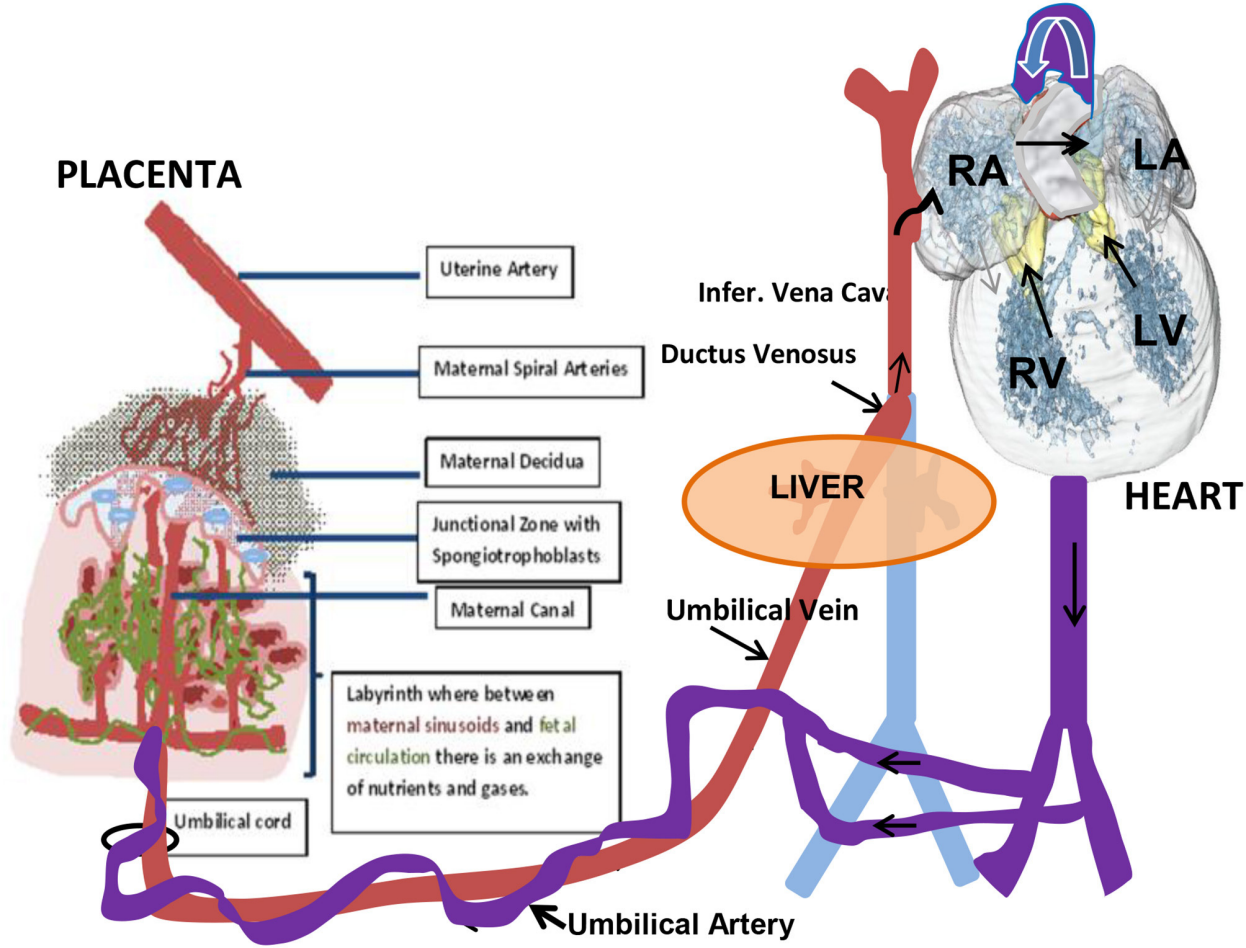
**Schematic diagram of the fetal-placental tissue organization and circulation**.

Although the gross anatomy of the human and mouse placenta are dissimilar in a number of ways, both share many cellular and molecular characteristics (Rossant and Cross, 2001). Briefly, the placenta is composed of both maternal and fetal tissues. The mouse placenta is composed of three layers: the maternal decidua that is of maternal origin uterine stromal cells across which maternal blood passes to the implantation site through spiral arteries; the junctional zone through which maternal blood vessels lead into and out of trophoblast giant cell-lined vascular spaces; and the labyrinth layer where nutrient and gas exchange occurs within a high surface area comprised of fetal and maternal vessels (Figure 2). A barrier, however, is maintained between the fetal and maternal circulations. The human placenta also has three similar layers: the maternal decidua layer with spiral arteries; a basal plate that is analogous to the junctional zone; and a labyrinth-like layer where the placental villi are organized into cotyledons, but is not as densely packed and the intervillous space is more open when compared to the rodent placenta.

#### Vascular remodeling and trophoblast invasion

Adequate utero-placental blood flow is necessary for normal pregnancy outcome. Vascular remodeling that takes place during utero-placental circulation provides sufficient blood flow throughout pregnancy. The vascular remodeling is mediated by trophoblast invasion of uterine spiral arteries, increased shear stress, and angiogenic and humoral biochemical factors (Osol and Mandala, 2009). What is unique to the placenta in both in human and rodent placentas is that the *maternal vasculature* is lined by *fetal cells* of the trophoblast lineage and not by maternal endothelial cells. Primary trophoblast cells that undergo invasion of the maternal blood vessels develop from the trophectoderm layer of the blastocyst (Rai and Cross, 2014). These primary trophoblast cells contribute to the formation of the decidua and promote the formation of maternal blood vessels that deliver blood to the placenta. These trophoblasts invade and assume the location and function of endothelial cells within the maternal vascular spaces in the placenta (Rai and Cross, 2014). The trophoblasts cells can also undergo morphogeneis forming vascular tubes de novo. The trophoblast invasion begins at approximately embryonic day (E) 8.5 in the mouse (in human pregnancy the first 10–12 weeks of gestation) (Rossant and Cross, 2001). The timing of invasion coincides approximately with the period that blood flow through the tubular heart first is detected in the mouse embryo (Phoon, 2001). After the first 10–12 weeks of human gestation as a result of the physiological change associated with trophoblast invasion, the diameter of the uterine spiral arteries increases, reducing impedance to flow. Abnormalities in the process can result in elevated resistance and pulsatility indices, which are associated with adverse maternal and offspring outcome.

The placental arterial tree in mice is supplied by a single umbilical artery, which branches into fetoplacental arteries and penetrates into the labyrinth region to the junctional zone (Figure 2). Perfusion of the arterial tree begins at E 9.5 as detected by flow in the umbilical artery (UA). Fetal growth is observed with increases in the diameter of the UA and in UA blood velocity that increases up to term at E 21 in the mouse. With the increase in diameters of the fetoplacental arteries there is a decrease in calculated vascular resistance of the arterial tree, that is similar to what has been described during human pregnancy (Mu and Adamson, 2006). This decrease in vascular resistance relates to the increased volume and surface area of the maternal blood spaces in the placental labyrinth of the mouse during pregnancy (Coan et al., 2004). Positive end-diastolic velocities are first detected at E15.5 and then increase to term. This coincides with the timing of the maturation of the four-chambered heart and placental development. It was shown that the PSV decreased between the maternal arterial canal and further decreased upon entering the labyrinth (Mu and Adamson, 2006). Low velocity in the terminal trophoblast-lined exchange spaces helps to promote the effective exchange of nutrients, gases, and waste products between the maternal and embryonic/fetal circulations.

#### Evidence for abnormal placental development and function with abnormal cardiovascular development

The link of abnormal placental development and function with abnormal cardiovascular development is suggested by several studies using animal models. Using the mouse model, the results from germ-line ablation of two specific genes, as *p38α-mitogen activated protein* (*MAP*) *kinase* or of *peroxisome proliferator-activated receptors (PPARs)*, demonstrated the above gene expression was critical for proper placental function and, when knocked out only in the placenta, resulted in cardiac defects. When the placental function was rescued, however, via aggregation with tetraploid embryos, the cardiac defects were rescued (Barak et al., 1999; Adams et al., 2000). In a study using the sheep animal model, using surgery before conception to reduce placental mass and induce fetal growth restriction, this resulted in an alteration of heart development in the sheep fetus (Morrison et al., 2007b). The placental restriction resulted in increased fetal hypoxia, intrauterine growth restriction (IUGR) and elevated plasmacortisol levels. There was no difference in relative heart weights between the control and placenta restricted fetuses. However the proportion of mononucleated cardiomyocytes increased. In relation to the relative size of the cardiomyocytes when expressed relative to heart weight, the cardiomyocytes were larger in the placenta restricted model in comparison to control fetuses. In this study effects on blood flow were not specifically analyzed and the cardiac effects may be due to an adaptation to the chronic hypoxia that resulted and hence the delay in binucleation and differentiation of the cardiomyocytes and a reduction in their number. Another study on sheep showed a similar link with IUGR and cardiomyocyte maturation and coronary artery function in fetal sheep (Bubb et al., 2007). It would appear these above-described myocardial changes may also induce changes in cardiac output that was not directly addressed. In general IUGR is not associated with development of cardiac defects. It is possible that in cases where embryos have severe hypoxia, IUGR, and heart defects, these embryos are not viable.

Effects of hypoxia on heart development were more directly addressed in quail embryos incubated under hypoxic conditions (16% oxygen) (Nanka et al., 2008). Their studies demonstrated thin ventricular walls developed and irregularities were observed in the development of the coronary tree. The hypoxia also resulted in increased capillarity and trabeculation. Subsequently, normal vascularization did not occur, leading to heart failure and embryonic death. For further discussion of effects of hypoxia on the fetus and heart development, a number of reviews have been published (Dunwoodie, 2009; Patterson and Zhang, 2010). In summary, studies suggest that possibly in the mammalian embryo a threshold of hemodynamic force, when present, allows normal development, but when below that threshold as a result of abnormal development, increasing severity of defects occur, or if the changes are very severe, the condition becomes lethal. The thresholds would need to be empirically determined.

## Studies from human gestation using doppler ultrasound data demonstrating changes in placental hemodynamics affecting cardiovascular development

### Failed trophoblast invasion and congenital heart defects

During early human pregnancy, before the intervillous space opens between 10 and 12 weeks of gestation (corresponding to ~16.6 days of mouse gestation), the development of the placenta in normal human pregnancies occurs as a “non-invasive trophoblast phenotype” in which the hypoxic environment prevents trophoblast invasion into the uterus that is mediated by cytokines as TNF alpha, IL-1 alpha, and 1-beta, VEGF and plasminogen activator inhibitor-1 (Caniggia et al., 2000). Subsequently, the extravillous trophoblast cells acquire “an invasive trophoblast phenotype” migrating into the uterine wall, replacing the maternal vascular wall, and transforming maternal spiral arteries from high resistance vessels into low-resistance vessels, allowing the entry of oxygenated and nutrient-rich maternal blood into the intervillous space. Failed trophoblast invasion and a high resistance have been described in women who subsequently deliver infants with growth restriction (Schulman et al., 1986). A recent report associates congenital cardiac defects with abnormal trophoblast invasion and abnormal placental angiogenesis (Llurba et al., 2014). In this case-control study of pregnant women carrying fetuses with major congenital cardiac defects, maternal venous blood, cord blood samples from fetuses with CHD and from non-CHD control fetuses were analyzed for angiogenic and anti-angiogenic factors. In termination-of- pregnancy cases, heart tissue was also analyzed from abnormal and normal fetal hearts. The study demonstrated the presence of hypoxia in abnormal human fetal hearts with an overexpression of HIF-2 alpha and HO-1. In the maternal serum of women carrying fetuses with major heart defects associated primarily with conotruncal and septal-valve defects, maternal serum placental growth factor (PlGF) was decreased and soluble fms-like tyrosine kinase-1 (sFlt-1) increased, suggesting impaired placental angiogenesis. A correlation between lower maternal PlGF levels and birth weight percentile in the CHD babies was observed suggesting that the placental impairment contributed to diminished growth potential of fetuses with CHD. In addition these fetuses demonstrated a trend toward a higher umbilical and lower middle cerebral artery Doppler resistance compared with controls. The authors concluded that evaluating the relationship between CHD and placental-related problems is an important area to pursue in future research.

### Umbilical blood flow and cardiac output

It is established that placental volume blood flow is a major determinant of early cardiac output, fetal growth, and well-being (Acharya et al., 2004, 2005; Vimpeli et al., 2009b). The blood flow through the umbilical cord corresponds to 17% of combined cardiac output at 10 weeks and 33% by 20 weeks (Kiserud and Acharya, 2004). The increase in umbilical flow optimizes fetomaternal gas and nutrient exchange and provides for healthy fetal outcomes. As seen in the Llurba et al. study, congenital malformations can be associated with hemodynamic changes occurring in the placenta and fetus, often resulting in smaller placentas and IUGR of the fetus. The circulatory effects may relate to the maturation of vascular responses in a number of organ systems (Rudolph, 2010): The effects of hemodynamic changes can lead to CHDs as well as affecting cerebral development in the fetus, as it has been measured that ~13% of the combined cardiac output between 11–20 weeks of gestation goes to the upper body, including the brain, during this period of pregnancy (Vimpeli et al., 2009a; Arduini et al., 2011). That cardiac and neural effects can be associated is suggested also by a report that provides evidence that children with CHDs are at increased risk of developmental disorders or developmental delay (Marino et al., 2012). The hemodynamic changes may severely affect neonatal survival or well-being, or when subtle, have long-term impacts relating to developmental disabilities or possibly adult cardiovascular disease. The latter relates to the Barker hypothesis regarding developmental origins of adult cardiovascular disease (Barker, 2007, 2008).

### Pregnant mothers with CHDs and effects on placenta and fetus

In the mammalian model abnormal cardiac development leading to congenital heart disease (CHD) has been associated with abnormal placental development with abnormal trophoblast invasion and blood vessel remodeling, abnormal blood flow, and the resultant changes in the transfer of nutrients and oxygen from the mother to the fetus. Impaired placental blood flow has been demonstrated also in pregnant women with CHDs and compromised heart function (Pieper et al., 2013) and was linked to the observed increased incidence of obstetric and offspring complications in the pregnancies of women with CHD. In this cited study it was analyzed whether preexisting cardiac dysfunction in the pregnant women with CHD can lead to abnormalities in placental development and abnormal utero-placental Doppler flow. The Doppler flow indices of the women with CHD indicated a higher resistance in the utero-placental circulation throughout pregnancy than seen in healthy women. Results showed that the abnormal utero-placental flow lead to increased IUGR and contributed to the observed pregnancy complications in comparison to control healthy pregnancies. Complications related to findings that more children of women with CHD were small for their gestational age (16.3 vs. 4.3% in control); congenital heart disease occurred in 4.8% of offspring of CHD vs. 0% of healthy women's offspring; offspring death occurred in 2.9% of the CHD group and 0% of the healthy group. Causes of death in the offspring related to spina bifida, complex heart disease, hydrops fetalis, and placental insufficiency.

Dysfunction of placental flow, as is seen to occur in human IUGR fetuses, leads to changes in intra-fetal cardiac dynamics that result from alterations in the preload, afterload, ventricular compliance and myocardial contractility (Kiserud et al., 2006). For example, the increase in placental and aortic (isthmus) impedance caused by vasoconstriction resulted in an increase in the right ventricular afterload. Subsequently, to adapt to hypoxemia, the vasodilatation of such vital organs as the brain, heart, adrenals and spleen occurred. Cerebral centralization and the increase of the ductus venosus flow also contributed to a greater increase in the preload of the right ventricle (RV) and to a preferential flow to the left ventricle (LV) that was associated with decreased LV compliance. As the placental resistance progresses and the cardiac overload increases, the coronary perfusion can lose its compensatory ability, leading to myocardial cell damage with decreased contractility and reduced cardiac output (Baschat et al., 1997; Crispi et al., 2008). The latter two effects were significantly correlated with increased perinatal morbidity and mortality. As observed in human and mouse pregnancy, abnormalities in utero-placental and umbilical arterial hemodynamics are associated with placental abnormalities and adverse cardiovascular outcomes.

## Hemodynamic changes and cardiac defects in the mouse model with environmental exposures

### Cross-talk between placenta and cardiovascular systems

Our results with the C57Bl6 mouse model demonstrated that a single environmental exposure occurring during gastrulation (on E6.75 of mouse gestation; extrapolated to human pregnancy ~ between 16 and 19 days after fertilization), many different forms of cardiac birth defects can arise. The experimental paradigm in our studies was to expose mouse embryos by a single intraperitoneal (i.p.) injection to the environmental factor at 6 PM on embryonic day E 6 (i.e., E 6.75) that corresponds to gastrulation during which cardiac and placental cell fate decisions are being made. Similar cardiac structural and functional defects in the mouse embryos resulted with the one-time exposure to the drug lithium (Li^+^), alcohol (EtOH), or homocysteine (HCys), the latter a natural metabolite and a marker of folate^1^ deficiency (Han et al., 2009; Serrano et al., 2010). With this gestational timing of exposure, we observed chiefly heart outflow related defects, valve defects (semilunar- and pulmonary-valve related), myocardial wall thickness changes, and changes in myocardial contractility. The embryo displayed IUGR and smaller placentas in comparison to control embryos. The data from the exposure studies led to the concept that both organ-systems, cardiac and placental, are important during cardiovascular development, including in the formation of the more mature four-chambered heart. It was evident that cell and molecular changes occurred in both the heart and placenta organ-systems after exposure and affected blood flow. There appears to be continuous cross-talk between the two organs via blood flow forces and biochemical signaling to adapt one's function to the needs of the other, and specifically, for growth of the embryo. Our data demonstrated both organ systems are affected by the exposures and one cannot say which comes first. Both adapt to the changes that each organ detects, but if the alterations that have taken place due to the exposures are severe enough, it will lead increasingly to compromised function and subsequent abnormal tissue and organ development, and possible death of embryos severely affected.

Abnormal branching of the fetoplacental arterial tree seems to take place both in human pregnancies and in mouse gestation where fetal growth restriction is evident with the umbilical artery hemodynamics compromised, as we reported with exposure of the pregnant mouse to the above-listed three environmental factors. Little is known about the mechanisms controlling the growth and the structure of the fetoplacental arterial tree. Although with environmental exposures, it is often disorganized and decreased vascularization is noted (Rennie et al., 2014).

### Changes in umbilical artery pulsatility index and resistance indices summarized

Umbilical artery flow is indicative of blood moving from the fetus to the placenta and the umbilical vein takes blood from the placenta to the fetus. The volume coming to the placenta, however, should be equal to volume going out of the placenta, as it is a closed system. There are strong clinical associations between abnormalities of umbilical artery hemodynamics, placental pathology and fetal growth restriction in the human. Although these parameters have been determined non-invasively by other investigators in the mouse using the Swiss-Webster mouse strain (Phoon et al., 2000) and the outbred CD-1, Harlan Sprague Dawley mouse (Mu and Adamson, 2006), because of possible strain differences (Rennie et al., 2012), we determined from values in our published data the normal umbilical artery (UA) blood flow patterns for the C57Bl6 fetal E15.5 mouse used in our exposure studies and compared with the control embryos exposed to physiological saline only by i.p. injection, and with embryos exposed by i.p. injection to the experimental environmental factors (Tables 1A,B). The trends and parameters of the control embryos were similar to those previously reported by others using the different mouse strains, although differences were noted between our controls receiving an injection and those that were uninjected (Han et al., 2009).

**Table 1A:** **Comparison of UA RI on E 15.5**.

**Treatment**	**UARI**
Hcys^a^ (*n* = 30)	0.73
Lithium^b^ (*n* = 23)^*^	0.87
EtOH^c^ (*n* = 26)^*^	0.93
Control^d^ (*n* = 24)	0.7

^a,b,^ and ^c^ compare with ^d^ separately to get p-value;

* *p < 0.000005, highly significant*.

**Table 1B:**
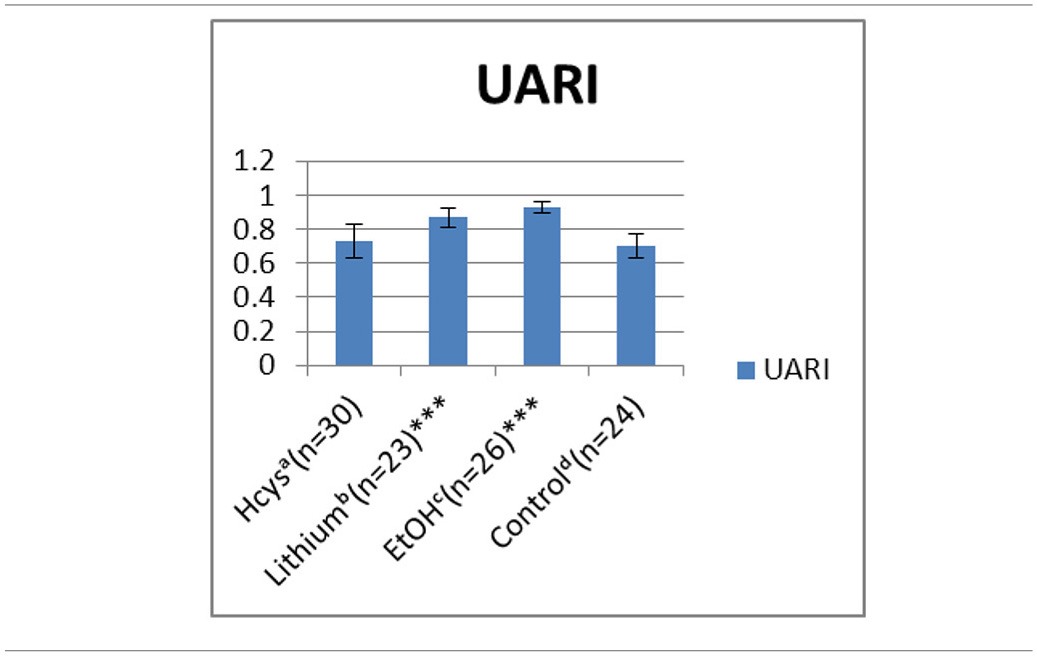
**Comparison of UA RI on E15.5**.

In human pregnancy, Doppler analysis of the umbilical circulation during second and third trimesters of pregnancy has proven to be a good predictor of fetal status. The significance of an abnormal umbilical Doppler in the first trimester has not been well established to be a predictor of early human pregnancy complications. Pulsatility index (PI) formula for the umbilical artery is the same as for the uterine artery, but in human pregnancy the EDV is not calculated for early stages, since it does not appear until 10 weeks of pregnancy, when measurements are only then attainable. It is similar in mouse gestation where EDV was very low in the umbilical artery before E14.5; detection rate was 38% at E 15.5 and 94% at E 18.5 (Mu and Adamson, 2006).

**Figure 3:**
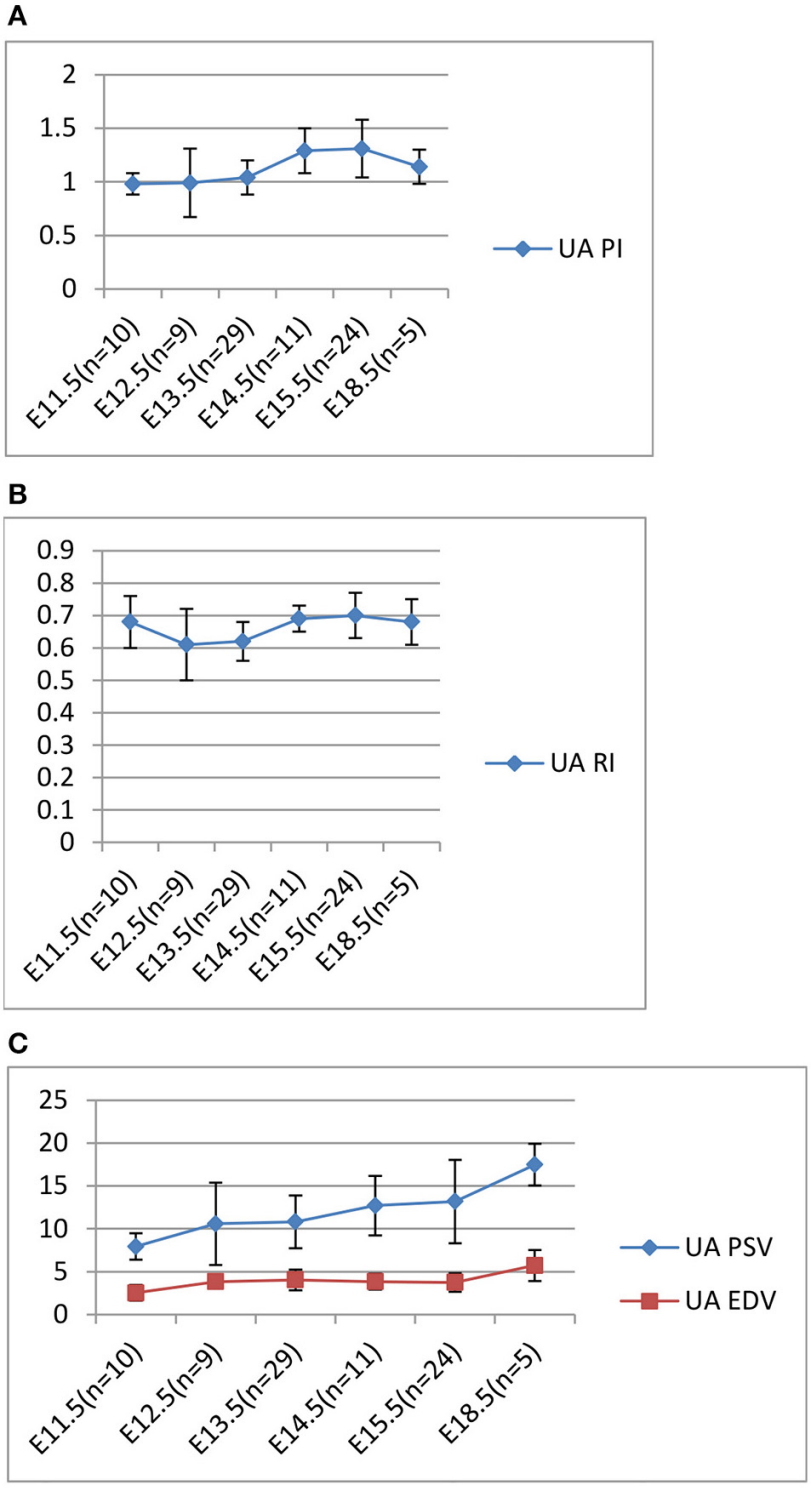
**Longitudinal Analysis: Doppler ultrasound parameters from E11.5 to E18.5 during mouse gestation. (A)** Umbilical artery (UA) Pulsatility Index (PI); **(B)** Umbilical artery (UA) resistance index (RI); **(C)** Umbilical artery (UA) PSV and EDV.

In our longitudinal study of the normal C57Bl6 mouse (uninjected) pregnancy, Doppler ultrasound measurements were carried out daily and the UA, PI, RI, systolic and end-diastolic peak velocities were calculated. To summarize these results, as well those of others, the PI increased from a low of 0.98 on E11.5 to 1.31 on E 15.5 and decreased to 1.1 on E 18.5 (Figure 3A). The RI results showed a similar pattern to the PI (Figure 3B). The systolic peak velocity (PSV) in umbilical artery increased with gestational age, significantly after E 15.5 (Figure 3C). The end-diastolic wave (EDV; Figure 3C) could be detected on E 11.5 and increased after E 15.5, concomitantly with time of PI and RI decreasing. These control mouse findings are similar to that described previously (Mu and Adamson, 2006).

Comparing the normal control pregnancy RIs (0.70) with the UA RIs of environmentally exposed E 15.5 embryos (Tables 1A,B): lithium (Li, 0.87) and ethanol (EtOH, 0.93) exposures significantly increased the RIs. With HCys exposure (0.73) the UA RI did not change significantly as compared to the control. The UA PI pattern, reported earlier, was similar to the RI and is not shown.

In summary, an elevation of HCys generally did not have a significant effect on placental development and blood flow, as did exposure to ethanol and lithium. This is reflected also in the effects on heart physiology. As published earlier, HCys displayed the best outcome of heart structure and function, followed by the poorer outcome with lithium's effects and with the most severe effects observed with alcohol exposure (Han et al., 2009; Serrano et al., 2010; Han et al., 2012).

Acute alcohol exposure during gastrulation induced the most severe effects. Alcohol induced abnormal valve formation, poor myocardial performance and abnormal placental development in more than 86% of embryos. Intraembryonically, echo cardiac parameters showed a significantly decreased cardiac cycle length, increased heart rate, altered IRT and ICT percentages, increased myocardial performance index (0.47 in control to 0.62 in alcohol exposed) reflecting increased afterload, and a decreased outflow velocity (36.07 in control decreasing to 26.73 with alcohol exposure) (Serrano et al., 2010). In an OCT study using an avian model, alcohol exposure was also demonstrated to alter early cardiac function and the functional changes preceded and were hypothesized to contribute to the observed late-stage valve and septal defects (Karunamuni et al., 2014).

In the future, it would be interesting to use these environmentally induced changes in cardiac function and the observed changes in the quantitative parameters of yolk sac and utero-placental blood flow to model possible biophysical effects of these flow alterations over time during abnormal cardiac morphogenesis.

### Morphometric parameters change with altered hemodynamics induced by environmental exposures

#### Lithium, homocysteine, and alcohol effects

IUGR is associated with placental and heart development (Corstius et al., 2005; Morrison et al., 2007a). Both Wnt/β-catenin signaling and homocysteine have been associated with placental development (Kamudhamas et al., 2004; Mohamed et al., 2008) and as we and others have shown with heart development (Rosenquist et al., 1996; Foley and Mercola, 2005; Manisastry et al., 2006; Ueno et al., 2007; Chen et al., 2008; Han et al., 2009). In all experimental groups, morphological parameters remained significantly altered. The morphometric data based on our published studies (Han et al., 2009; Serrano et al., 2010) are summarized in Table 2. As with Li and HCys exposures, the affected EtOH-exposed embryos displayed significant intrauterine growth retardation (body weight, BW) and smaller placental weight (PW). Peripheral Doppler changes included increased placental resistance and possibly increased lower body systemic resistance.

**Table 2:** **Morphological Parameters to the EtOH, Lithium, and HCys treated E15.5**.

	**CRL (mm)**	**BW (gm)**	**PW (gm)**
Normal control (*n* = 24)	15.33	0.44	0.13
EtOH (*n* = 26)	12.85^***^	0.39^*^	0.10^***^
Li (*n* = 23)	13.35^***^	0.36^***^	0.11^**^
HCys (*n* = 30)	13.1^***^	0.36^***^	0.11^***^

Each treatment group vs. normal control (NC) group was calculated to get a probability (p) value (t-test): A p-value < 0.05 was considered significant;

* p < 0.05;

** p < 0.0005;

*** *p < 0.000005*.

These morphometric changes in embryonic and placental size may relate to the above-mentioned altered vascularity (Reynolds et al., 2006). The compromised vessel development seemingly is due to the exposure effects decreasing hematocrit and viscosity (Lucitti et al., 2007), and reducing trophoblast invasion of the maternal blood vessels. All three mechanisms may be involved.

#### Cigarette smoke effects

A similar reduced vascularization of the fetoplacental arterial tree has been shown to occur with embryonic exposure to cigarette smoke toxins using micro-computed tomography (Rennie et al., 2014). In fetal sheep, the intraplacental arteries, arterioles, capillaries, and venules of the arterial tree represent ~55% of resistance across the fetoplacental circulation with the rest primarily based in the umbilical artery (Adamson et al., 1989). Our data reflecting smaller placentas that were observed with our environmental exposures and the possible compromise of trophoblast invasion of the arterial bed would suggest the development of a compromised vascular system: We demonstrated using the human trophoblast cell line HTR-8/SVneo studies that trophoblast migration is decreased with environmental exposures, but not cell proliferation (Han et al., 2012). This would result in poor trophoblast invasion of the spiral arteries and ultimately relate to poorer exchange of nutrients, gases, and waste products. It would be expected that these changes would facilitate the development of intrauterine growth restricted embryos and the CHDs that were observed and also the increase in numbers of resorbed embryos that we reported with the three experimental exposures.

We have reported in the mouse model multiple cellular-molecular changes that take place in the mammalian placenta with environmental exposures. The labyrinth appears disorganized with a thinner maternal decidua apparent in the smaller placentas in comparison to control placentas (Han et al., 2012). As based upon some of the above described studies, it is expected that reduced and disorganized vascularization would relate to the poor myocardial performance, alterations of intraembryonic hemodynamic blood flow forces, and to the CHDs, including of the valves, that we reported (Chen et al., 2008; Han et al., 2009). In our analysis of one cardiac cycle of the embryonic avian heart, the highest blood flow forces and cardiac jelly displacement related to the region that subsequently gives rise to the endocardial cushions during valve development (Garita et al., 2011). Similar conclusions in regards to impact of changes in hemodynamic forces on valve development are reached by investigators in this volume, as well as by others (Hogers et al., 1999; Butcher and Markwald, 2007; Vermot et al., 2009; Tan et al., 2011; Peterson et al., 2012).

## Conclusion

In conclusion, with poor vascularization and reduced trophoblast invasion, hemodynamics of flow to the embryonic heart is altered and heart function is compromised. In turn in a closed system, the decreased cardiac contractility would lead to decreased flow forces in the umbilical artery returning blood to the placenta. It appears there are continuous adaptive changes in both the developing heart and in the placenta to accommodate the embryo's needs as it grows. In response to the physiological effects of the environmental insults, changes in flow forces are detected in the cross-talk between the respective organs, and adaptive changes to normalize development may no longer be met and increasingly lead down the path toward CHDs.

As seen in this Special Topics issue and in previous studies, we now have ample evidence that biomechanics and blood flow parameters are of critical importance in heart and vascular morphogenesis and growth of the embryo and fetus. The hemodynamic studies that have been carried out in mouse and human pregnancy, and the environmental studies in animal models suggest that an important component that needs to be taken into consideration in the mammalian model is the yolk sac and placental blood flow. With use of avian, zebrafish, and transgenic mouse models in which gene expression can be conditionally modulated to target either the heart or placenta, these future studies will enable us to better understand the contribution of extraembryonic blood flow and specific blood flow parameters necessary for normal embryonic/fetal cardiovascular development and those that will lead to dysmorphogenesis. It will be possible to use the quantitative measurements on flow changes in the modeling of biophysical forces and effects on subsequent cardiac structural development in order to define more precisely those threshold hemodynamic force changes that lead to the different types of anomalies that are observed.

### Conflict of interest statement

The authors declare that the research was conducted in the absence of any commercial or financial relationships that could be construed as a potential conflict of interest.

## Footnotes

^1^*Dietary Supplement Fact Sheet: Folate*. National Institutes of Health, Office of Dietary Supplements. Available online at: http://ods.od.nih.gov/factsheets/Folate-HealthProfessional/
